# Integrating multiple land cover maps through a multi-criteria analysis to improve agricultural monitoring in Africa

**DOI:** 10.1016/j.jag.2020.102064

**Published:** 2020-06

**Authors:** A. Pérez-Hoyos, A. Udías, F. Rembold

**Affiliations:** European Commission, Joint Research Centre (JRC), Via E. Fermi 2749, I-21027 Ispra, VA, Italy

**Keywords:** Multi-criteria analysis, Data fusion, Land cover/land use, Agriculture mask, Agriculture monitoring

## Abstract

Monitoring agricultural land cover is highly relevant for global early warning systems such as ASAP (Anomaly hot Spots of Agricultural Production), because it represents the basis for detecting production deficits in food security assessment. Given the significant inconsistencies among existing land cover datasets, there is a need to obtain a more accurate representation of the spatial distribution and extent of agricultural area in Africa. In this research, we explore a fusion approach that combines the strength of individual datasets and minimises their limitations. Specifically, a semi-automatic method is developed, relying on multi-criteria analysis (MCA) complemented with manual fine-tuning using the best-rated datasets, to generate two hybrid and static agricultural masks – one for cropland and another for grassland. Following a comprehensive selection of land cover maps, each dataset is evaluated at country level according to five criteria: timeliness, spatial resolution, comparison with FAO statistics, accuracy assessment and expert evaluation. A sensitivity analysis is performed, based on an evaluation of the impact of weight settings on the resulting land cover. The proposed methodology is capable of improving agricultural characterisation in Africa. As a result, two static masks at 250 m spatial resolution for the nominal year 2016 are provided.

## Highlights

• A multi-criteria analysis (MCA) is developed to generate a cropland and grassland hybrid mask.
• Each land cover dataset is evaluated at country-level by five different criteria.
• The MCA approach offers a flexible and transparent methodology to combine different data.
• The hybrid masks are used in the JRC-ASAP early warning system and are freely accessible.

## Graphical abstract

**Figure:**
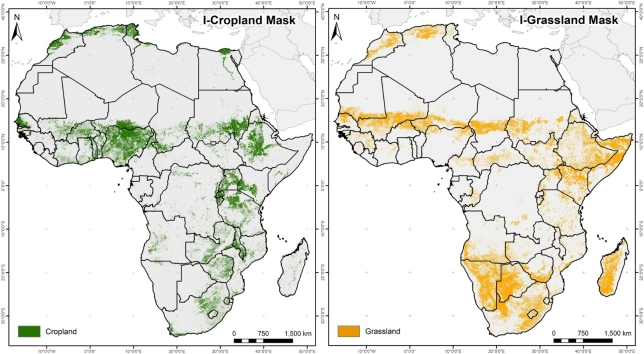

## 1 Introduction

Reliable and accurate mapping of the extent and location of agriculture plays an important role in a series of applications, such as yield estimation, rural development and food security assessment (Fritz et al., 2019). Resulting maps provide basic inputs for monitoring agriculture in early warning systems such as the recently launched Anomaly hot Spots of Agricultural Production (ASAP) (https://mars.jrc.ec.europa.eu/asap/wexplorer/) (Rembold et al., 2019). This system requires land cover/land use information, suitable not only for crop, but also for livestock production (FAO, 2014). It is also crucial to monitor grassland in Africa, where it covers roughly half of the continent and plays a fundamental role in population food supply (Holechek et al., 2017).

Remote sensing significantly contributes to efficient monitoring of the spatial distribution of agriculture; either as a single class (i.e. cropland) or as part of a multi-class land cover classification (Pittman et al., 2010). Representative land cover products at medium and low resolution include Global Land Cover 2000 (GLC2000) (Mayaux et al., 2006), MODIS Land Cover (MODISLC) (Friedl et al., 2010), GlobCover (Bicheron et al., 2008), ESA Climate Change Initiative Land Cover (CCI-LC) (Bontemps et al., 2009) and Global Land Cover by National Mapping Organizations (GLCNMO) (Tateishi et al., 2014). Alternatively, specific cropland maps have been developed, such as the Global Map of Rainfed Cropland Areas (GMRCA) (Birandar et al., 2009), Global Irrigated Area Map (GIAM) (Thenkabail et al., 2009) or agricultural map produced by Ramankutty et al. (2008). However, their low spatial resolution at 10 km or limited accuracy restrict their operational application at national and regional level (Vancutsem et al., 2013). Additionally, a plethora of regional and national products has been produced, such as Africover (FAO, 1996), developed at country level and in higher resolution. Nonetheless, it is hampered by its low update frequency and limited geographic coverage.

ESA’s recent Sentinel images, together with the free availability of the Landsat archive (Wulder et al., 2012) allow a move towards a new era of higher-resolution (e.g. 20 m) land cover mapping (Herold et al., 2016). Furthermore, the development of new cloud technologies and processing tools, such as Google Earth Engine, allows the processing of big data for scientific purposes and enhances capability for working at higher spatial resolution for large areas. Products based on such technologies include the 30 m GlobeLand30 (Chen et al., 2015), newly released CGLS (Copernicus Global Land Service) dynamic Land Cover map (Buchhorn et al., 2017), ESA CCI S2 prototype Land Cover map (Lesiv et al., 2017) and GFSAD (Global Food Security-Support Analysis Data) project (Xiong et al., 2017).

Despite the existence of a wide range of products, prior research highlights considerable disparity between them, especially when considering agricultural classes (Fritz et al., 2013). Hence, their quality remains uncertain and their use for a specific application is still questionable (Chen et al., 2015). Particularly for Africa, where widespread disagreement is found among datasets (Tsendbazar et al., 2015) and only 2.5 % full agreement was found for cropland when comparing nine global land cover datasets (Pérez-Hoyos et al., 2017).

Hybridisation or data fusion, defined as the process of integrating existing land cover datasets into a single one, represents a worthy approach to reconciling the strengths of individual sources and overcoming some of their main limitations (Herold et al., 2016). First attempts have relied on a voting procedure with an agreement scoring method, due to its simplicity and operability (Lu et al., 2017). Jung et al. (2006) used this approach to develop a global land cover map for carbon cycle modeling, based on a fuzzy agreement scoring method. Similarly, Pérez-Hoyos et al. (2012a,Pérez-Hoyos et al., 2012b developed a hybrid land cover map for Europe from a probability-voting scheme that reconciles legends based on a fuzzy agreement score and includes the probability of each class being the correct one. Another methodology broadly used for data integration relies on expert knowledge, such as the one used to derive a cropland map of Africa adopting expert knowledge to rank five land cover products (Fritz et al., 2010). The methodology was further enhanced by including a quantitative validation by experts to produce a 1 km hybrid percentage cropland product at global scale (Fritz et al., 2015). Similarly, Vancutsem et al. (2013) harmonised different datasets to derive a crop mask for Africa using an expert-based approach, while Latham et al. (2014) applied rules defined in the FAO Land Cover Classification System (LCCS) to derive the FAO Global Land Cover – SHARE (GLC-SHARE) datasets.

Spatial analytical and statistical approaches are becoming increasingly popular. For example, Schepaschenko et al. (2015) used geographically weighted regression (GWR) to derive a global hybrid forest map, whilst See et al. (2015a) applied it to derive a global land cover map. Lesiv et al. (2016) compared GWR with other methods (nearest neighbour, Naive Bayes and regression trees) to derive a hybrid forest cover map. More recent methodologies rely on more sophisticated algorithms, such as regression-kriging (Tsendbazar et al., 2016), decision trees (Yu et al., 2013), machine learning (Song et al., 2017), Bayesian spatial statistical approach (Gengler and Bogaert, 2018) and Dempster-Shafer theory (Song et al., 2014a,b). Recently, Waldner et al. (2016) effectively used a multi-criteria decision-making (MCDM) method to derive a cropland map.

This research is the extension of a preliminary study (Pérez-Hoyos et al., 2017) where, after a thorough comparison of nine global land cover datasets, we found strong disagreement among them, particularly in Africa. This fosters the hypothesis that, despite advances in agricultural mapping in recent years due to refinement of methodologies and availability of new datasets, there is still a need to ascertain the exact dimension of agricultural areas and their spatial distribution. Hereafter, the overarching goal of this work is to derive two static hybrid agricultural maps at 250 m spatial resolution, one for cropland and another for grassland (hereafter referred to as I(Integrated)-Cropland and I-Grassland) for the nominal year 2016 in Africa. In order to fulfil this objective, a semi-automatic hybrid methodology relying on multi-criteria analysis is proposed and further tested. To evaluate the performance of the method, the resulting agricultural masks are compared with existing hybrid datasets, and their accuracy assessed based on independent validation samples.

## 2 Datasets

A comprehensive review has been made of the most commonly used land cover datasets, and the most recent and highest resolution datasets have been analysed.

### 2.1 Land cover datasets

The datasets used in this study are listed in Table 1. More details of their specific characteristics can be found in the references provided in the table. The datasets are completely free and publicly accessible on the web, except for a limited number that are available only upon request. The datasets are split into two groups: 1) global or continental data, and 2) regional or national data.

**Table 1:** Summary of global land cover datasets analysed to develop the I-masks.

Data Source	Reference	Spatial resolution	Sensor	Data acquisition	Extent
**GLOBAL AND CONTINENTAL**					
**CGLS-LC100 V1.0 (Copernicus Global Land Cover)**	(Buchhorn et al., 2017) http://land.copernicus.vgt.vito.be/PDF/datapool/Vegetation/Dynamic_Land_Cover/Land_Cover_100m_Africa_V1_NETCDF/	100 m	PROBA-V	2015	Africa
**GLC2000 (Global Land Cover) - JRC**	Bartholomé and Belward (2005) http://forobs.jrc.ec.europa.eu/products/glc2000/products.php	1 km	SPOT 4 –VGT	Nov 1999-Dec 2000	Global
**GLCNMO V2 (Global Land Cover by National Mapping Organizations) - ISCGM**	Tateishi et al. (2014) https://globalmaps.github.io/glcnmo.html	500 m	MODIS	2008	Global
**GlobCover 2009 - ESA**	Bicheron et al. (2008) http://due.esrin.esa.int/page_globcover.php	300 m	MERIS FR	2009	Global
**GlobeLand30 - UN/NASG**	Chen et al. (2015) http://www.globallandcover.com	30 m	Landsat TM, ETM7, HJ-A1/B	2010	Global
**LC-CCI 2015 (Land Cover Climate Change Initiative) - ESA**	CCI (2017) http://maps.elie.ucl.ac.be/CCI/viewer/download.php	300 m	MERIS FR/ PROBA-V/SPOT-VGT/AVHRR/HRPT	2015	Global
**MODISLC 2010 - NASA**	Friedl et al. (2010) https://lpdaac.usgs.gov/dataset_discovery/modis/modis_products_table/mcd12q1	500 m	MODIS	2010	Global
**ESA CCI Land Cover S2 prototype**	http://2016africalandcover20m.esrin.esa.int/download.php	20 m	Sentinel-2A	December 2015- December 2016	Africa

***REGIONAL AND NATIONAL***					
**Africover - FAO**	Di Gregorio and Jansen (1996) http://www.fao.org/geonetwork/srv/en/main.search?title=africover	30 m	Landsat 7	1995-2002	Burundi, Democratic Republic of Congo, Egypt, Eritrea, Kenya, Rwanda, Somalia, Sudan, Tanzania and Uganda
**Congo Basin - UCL**	Verhegghen et al. (2012) https://maps.elie.ucl.ac.be/	300 m	MERIF FR/ SPOT VGT	December 2004- June 2006	Cameroon, Congo, Gabon, Burundi, Central African Republic, Equatorial Guinea, Republic Democratic of Congo and Rwanda
**CORINE**	Jaffrain (2011)	100 m	Landsat	1990	Morocco, Tunisia
**Democratic Republic of Congo - UCL**	Vancutsem et al. (2009) http://maps.elie.ucl.ac.be/geoportail/	1 km	SPOT- VEGETATION	2000	Democratic Republic of Congo
**GLCN (Global Land Cover Network) - FAO**	http://www.fao.org/geonetwork/srv/en/main.home	30 m	Landsat-8	2005-2014	Kenya, Malawi, Senegal, South Sudan, Sudan, Tunisia
**SERVIR – NASA/USAID** **Land Cover Scheme I and II**	https://www.servirglobal.net/Global/Maps-Data/Data-Catalog	30 m	Landsat	2008-2014	Botswana, Ethiopia, Lesotho, Malawi, Namibia, Rwanda, Tanzania, Uganda, Zambia
**Mozambique Land Cover- DNTF**		100 m	Landsat	2007	Mozambique
Repsahel (Amélioration de la résilience des populations sahéliennes aux mutations environnementales) – OSS*	http://www.oss-online.org/rep-sahel/index.php?lang=en	30 m	Landsat 5, 8	2011-2013	Burkina Faso, Chad, Mali, Mauritania, Senegal, Niger
**SADC (Southern African Development Community) -CSIR**	http://gsdi.geoportal.csir.co.za/projects	30 m	Landsat 7	1990-1995	Lesotho, Malawi, Mozambique, South Africa, Swaziland, Tanzania, Zimbabwe
**SANBI**	http://bgis.sanbi.org/landcover/project.asp	30 m	Different land covers	2009	South Africa
**South Africa Land Cover**	http://bgis.sanbi.org/DEA_Landcover/project.asp	30 m	Landsat 8	2013-2014	South Africa
**SWALIM (Somalia Water and Land Information Management project) - FAO**	http://www.faoswalim.org/land/land-cover	520 m	Landsat	2009-2011	Somalia
**West Africa Land Use Land Cover Time Series – USGS** **ECOWAS**	Tappan et al. (2016) https://eros.usgs.gov/westafrica/data-downloads	2 km	Landsat 8	2010-2013	Benin, Burkina Faso, Chad, Ivory Coast, Gambia, Ghana, Guinea, Guinea-Bissau, Liberia, Mali, Mauritania, Niger, Nigeria, Senegal, Sierra Leone, Togo
Woody Biomass - World Bank*		30 m	Landsat	2000	Ethiopia

* Available upon request.

### 2.2 Reference datasets

Traditionally, one of the major drawbacks in carrying out a validation is the limited availability of common reference datasets (Congalton et al., 2014. However, in recent years much effort has been dedicated to developing initiatives that facilitate free accessibility and availability of reference data. In this study, we exploit some of these – specifically, those allocated to land cover validation – by joining them into a single set composed of 5,058 samples see more details in Table 2) (Tsendbazar et al., 2016). Five ground reference datasets from GOFC-GOLD (Global Observations for Forest Cover and Land-use Dynamics) are used: GLC2000 (Bartholomé and Belward, 2005); GlobCover 2015 (Bicheron et al., 2008); STEP (System for Terrestrial Ecosystem Parameterization) (Friedl et al., 2010); VIIRS (Visible Infrared Imaging Radiometer Suite) (Olofsson et al., 2012); and GLCNMO 2008 training dataset (Tateishi et al., 2014). In addition to the GOFC-GOLD datasets, we also use data from the FROM-GLC project (Zhao et al., 2014) comprising point training samples. Furthermore, we used data from the validation carried out by experts from the International Institute for Applied Systems Analysis (IIASA) in development of the JRC crop mask (Vancutsem et al., 2013). Finally, the reference sample set has been enlarged with samples from the second Geo-Wiki competition, carried out from January to March 2012 (Comber et al., 2013).

**Table 2:** Summary of reference datasets used in this study.

Source	Validation datasets	Geometry	Legend	Legend
			Cropland	Grassland
GOFC-GOLD http://www.gofcgold.wur.nl/sites/gofcgold_refdataportal.php	GLC2000	Points	8. Cultivated and managed vegetation	7. Herbaceous vegetation
	GlobCover	5 × 5 MERIS pixels (225 ha)	11. Post-flooding or irrigated cropland 14. Rainfed cropland 20. Mosaic cropland/vegetation 30. Mosaic vegetation/cropland	120. Mosaic grassland/forest or shrubland 140. Closed to open herbaceous vegetation
	STEP	Polygon (4 × 4 km)	12. Croplands 14. Cropland/natural vegetation	9. Savannas 10. Grasslands
	VIIRS	Polygon (5 × 5 km)		
	GLCNMO	Polygon	11. Cropland 12. Paddy field 13. Cropland/Other vegetation	8. Herbaceous 9. Herbaceous with sparse
Geo-Wiki https://www.geo-wiki.org/downloads/	IIASA experts	Polygon (1 × 1 km)	% of cropland	__
	Competition II	Polygon (1 × 1 km)	4. Cultivated and management/cropland 5. Mosaic cultivated and management	3. Herbaceous vegetation/grassland
Zhao et al. (2014) http://data.ess.tsinghua.edu.cn/	FROM-GLC	Point	10. Cropland	30. Grassland

## 3 Methods

**Fig. 1:**
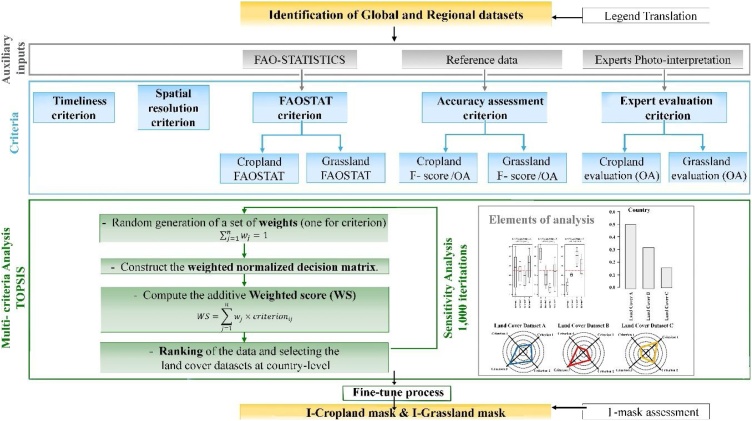
Flowchart summarising the main steps in the methodology.

The general outline of the method includes the following steps (Fig. 1).

1 Identification, collection and pre-processing of land cover dataset (3.1).
2 Legend translation and harmonisation (3.2).
3 Multi-criteria analysis (MCA) (3.3).
  - Identification of evaluation criteria and assessment of each dataset according to these (3.3.1). The five criteria that fulfil the requirements for a land cover map (Waldner et al., 2015) and affect map accuracy (Fritz et al., 2013) are timeliness, spatial resolution, comparison with FAO statistics, accuracy assessment and expert evaluation.
  - Construction of standardised performance matrix for each country (3.3.2).
  - Sensitivity analysis and ranking (3.3.3).
4 Fine-tune approach performed by human intervention among the upper-ranked alternatives, for those countries where there is no clear winner through MCA.
5 Composition of the two hybrid masks by combining the most appropriate land cover for each country.
6 Accuracy assessment of the resulting hybrid mask (3.4).

### 3.1 Data pre-processing

The initial step involves co-registering and re-projecting all data to a comparable geographic projection and resolution. The datasets are thus re-projected onto the WGS 84 (World Geodetic System) latitude/longitude, and re-sampled to a spatial resolution of 250 m (0.00225 degrees) using an average resampling method (Jung et al., 2006; Chen et al., 2017), as this is a compromise resolution for monitoring agriculture at the national and regional level with medium resolution time series (Pérez-Hoyos et al., 2017). Those datasets that are originally in vector format (e.g. Africover) are rasterised at 250 m spatial resolution, using the maximum area criteria (Vancutsem et al., 2013).

### 3.2 Legend translation and harmonisation

One of the most controversial issues for this study is in defining cropland and grassland, as there is no consensus on one single definition. On the contrary, many different ones are proposed. Moreover, we face the problem of using land cover datasets to define land use classes needed for agricultural monitoring. Here, FAO (2014) terminology is implemented; this considers agricultural land as land suitable for livestock and crop production, which can be further split into arable land (i.e. land under annual crops or land temporarily fallow), land under permanent crops, and permanent pastures. Following a pragmatic definition in line with the datasets used, in this paper ‘cropland’ refers to arable land and permanent crops grouped together, as some data (e.g. GlobeLand30) make no distinction between them.

The definition of grassland entails even more complexity, as it includes a broad land use gradient ranging from natural grassland with tree cover, through sparse vegetation to intensively managed rangelands and pastures (Friedl et al., 2010). In this context, the separation between land cover and land use terms remains even more challenging than for crops (e.g. pasture versus grassland versus herbaceous vegetation) (Herold et al., 2008). At the same time, from a remote sensing point of view, clear distinction of grassland is difficult because this class is frequently confused with similar spectral classes, such as shrublands. Bearing in mind that the final scope of our I-Grassland is as a basis for assessing anomalies in forage availability (and ultimately livestock production) using remote sensing data, we use a conservative approach that minimises spectral variation due to possible presence of woody vegetation. Hence, more emphasis is allocated to herbaceous zones, whereas other classes that could also have a pastoral use (e.g. woody savannas or dense shrublands) are excluded from our grassland definition. In this framework, grassland is defined according to FAO GLC-SHARE terminology, which corresponds to: any geographic area dominated by natural herbaceous plants with a cover of 10 % or more, irrespective of different human and/or animal activities, such as grazing, etc. Woody plants (trees and/or shrubs) can be present assuming their cover is less than 10 % (Latham et al., 2014).

To overcome some of the limitations due to the heterogeneity of the products and differences in the classification system for each dataset, and to minimise ambiguities, all land cover classes are accommodated into a common or target legend composed of three classes (cropland, grassland and others). For legend translation, we have implemented the approach proposed by Herold et al. (2008), which uses the FAO LCCS as a conceptual framework (Di Gregori and Jansen, 2000). In fact, most of the products analysed already use LCCS in their development (e.g. GlobCover, Africover, DRC map, GLCNMO2008, GeoNetwork), simplifying the translation. Others do not, such as MODISLC which is based on the International Geosphere-Biosphere Programme (IGBP), or GlobeLand30 which adopts a ten-class classification scheme defined by Chen et al. (2015). In this case, the LCCS-based legend translation protocols proposed by GOFC-GOLD in tandem with the FAO/UNEP Global Land Cover Network (Herold et al., 2009), along a reference benchmark (e.g. Herold et al., 2008), are used to harmonise the legends. Moreover, to ensure consistency with the previously produced MARS-JRC, mosaic classes with a majority fraction (>50 %) are included in the masks, while for minority fractions (20–50 %) a thorough analysis is made through visual interpretation, using Google Earth high resolution imagery at country level, to determine whether they should be included in the class analysed. This is essential for equatorial countries where croplands are mixed with forest and only mosaic classes are used to describe agricultural areas, such as in Guinea where croplands are only mapped by class Mosaic forest/cropland according to GLC2000 (Vancutsem et al., 2013).

### 3.3 Multi-criteria analysis (MCA)

A semi-automatic method is proposed, relying on multi-criteria analysis (MCA) and fine-tuned among the best-rated land cover for each country, in order to create two hybrid agricultural masks for Africa (I-Cropland and I-Grassland). MCA is applied at country level in ranking the different options (i.e. land cover datasets), or making choices among the finite set of alternatives in a transparent and objective way, by taking five criteria into account (Keeney and Raiffa, 1993). The MCA implemented is based on a well-established TOPSIS (Technique for Order of Preference by Similarity to Ideal Solution) method that ranks alternatives according to their distance from a positive and negative ideal solution (Hwang and Yoon, 1981). The TOPSIS method encompasses the following steps. 1) Create a performance decision matrix based on a set of *n* criteria and *m* alternatives. 2) Standardise the matrix. 3) Compute the weights for each criterion and calculate the weighted normalised decision matrix. 4) Identify the positive and negative ideal solutions. 5) Calculate the distance of each criterion from the ideal positive and negative solutions. 6) Compute the closeness coefficient of each alternative. 7) Rank the alternatives.

The proposed MCA approach integrates a sensitivity analysis capable of identifying situations where one land cover performs clearly better than the rest, and those where a fine-tune process based on visual analysis is required. The sensitivity analysis is supported by partial results from the MCA, such as multiple boxplot, the ranking alternative and the radar diagrams. Furthermore, the analysis is only needed when two or three alternatives come out similarly, depending on the relative importance or weight assigned to each criterion.

#### 3.3.1 Identification of evaluation criteria

One of the key choices in development of the MCA is the identification of a compendium of adequate and representative criteria applied in evaluating the different land cover datasets at country level. After a comprehensive review of literature (e.g. Congalton et al., 2014; See et al., 2015a), five representative criteria are identified that fully cover the main requirements for an agricultural map, and that affect agricultural mapping accuracies (Waldner et al., 2015; Fritz et al., 2013).

##### 3.3.1.1 Timeliness criterion

Timeliness refers to the time interval between the production of a dataset and the target reference year for our hybrid mask (i.e. 2016). The premise is that timeliness influences accuracy and representativeness of data due to land cover changes in agriculture, such as conversions from natural vegetation to agricultural crops, urbanisation or agricultural abandonment (Schepaschenko et al., 2015). Therefore, to minimise the impact of these changes, the most recent products are favoured through a logarithmic interpolation (Eq. (1)) that ranges from 1 (i.e. nominal year of the output mask – 2016) to 0 (i.e. oldest dataset – 1992). The logarithmic interpolation tends to favour the target year and adjacent years.

(1) Timeliness Criterion=83.499  ln(year)-634.333

##### 3.3.1.2 Spatial resolution criterion

Spatial resolution refers to the smallest land feature to be detected and consequently mapped (FAO, 2017). In Africa, where agricultural field size is generally small, with low agricultural mechanisation (Pittman et al., 2010), a fragmented and heterogeneous landscape, and a spatial distribution more scattered than other cropping regions in the world (Yadav and Congalton, 2018), higher spatial resolution can better characterise agriculture (Lobell and Asner, 2004). Hence, higher priority is given to the most spatially detailed dataset, through a logarithmic interpolation (Eq. (2)) where data range from 1 (highest spatial resolution of 20 m) to 0 (lowest spatial resolution of 2 km).

(2) SR Criterion=-0.217  ln(Spatial Resolution)+1.65

##### 3.3.1.3 FAOSTAT criterion or comparison with statistics

This criterion refers to consistency with agricultural statistics at country level. Data on agricultural extent and productivity are available through the Food and Agriculture Organization of the United Nations (FAO), which collects statistics from countries and harmonises it to form a global database. Although it is recognised that accuracy among countries is heterogeneous, the dataset is the most comprehensive agricultural database, mainly in regions where the availability of statistics is a challenge as occurs in many African countries (FAO, 2012).

Statistics from FAOSTAT (http://faostat3.fao.org/home) are used and averaged from 2006 to 2016 to reduce the effects of interannual variability. The total area of cropland and grassland is compared with the statistics, using the sum of ‘arable land’ and ‘permanent crops’ for cropland (i.e. Cropland FAOSTAT in Fig. 1) and ‘permanent meadows and pastures’ for grassland (i.e. Grassland FAOSTAT). The criterion is based on the absolute difference of statistics from the different land cover datasets available at country level (see Pérez-Hoyos et al., 2017 for more details).

According to the data provided by FAOSTAT, a factor that computes the proportion of surface allocated to cropland or grassland, compared with the total surface of the country, normalises this criterion and the ones described below. In this way, the effect of an omitted class is minimised, such as in Egypt where the presence of grassland is almost negligible. Hence, this class is normalised around zero for this particular case.

##### 3.3.1.4 Accuracy assessment criterion

Map accuracy assessment is a key component of map production as it provides a quantitative description of the quality of the land cover dataset (Strahler et al., 2006). To compare the accuracy of the different land cover datasets, a common accuracy assessment is carried out at country level, based on the reference data described in subsection 2.2.

To minimise problems associated with the process of validation itself, the analysis is only conducted in the areas classified as croplands or grasslands by at least one of the datasets analysed. Otherwise, the unbalanced surface of cropland or grassland, in proportion to the surface occupied by other classes, would result in a false overestimation of the metrics derived from the validation. The accuracy assessment is then executed by means of the widely accepted confusion or error matrix, which is a simple cross-tabulation of the classified dataset against the reference data described above (Congalton, 1991). Some common metrics are derived from this, including overall accuracy (OA), user accuracy (UA) (or commission error), and producer accuracy (PA) (or omission error) (Foody, 2002). Furthermore, a more informative accuracy metric such as the F-score is calculated as a combination of the PA and UA for cropland and grassland classes, leading to the following equation:

(3) $${\text{F}}_{\text{s}\text{c}\text{o}\text{r}\text{e}}=2\times \frac{\text{U}\text{A}\times \text{P}\text{A}}{\text{U}\text{A}+\text{P}\text{A}}$$

The final parameters used in this criterion are the overall accuracy as a global evaluation of each land cover and two types of specific indices, the F-score cropland and F-score grassland (see Fig. 1).

##### 3.3.1.5 Expert evaluation criterion

**Fig. 2:**
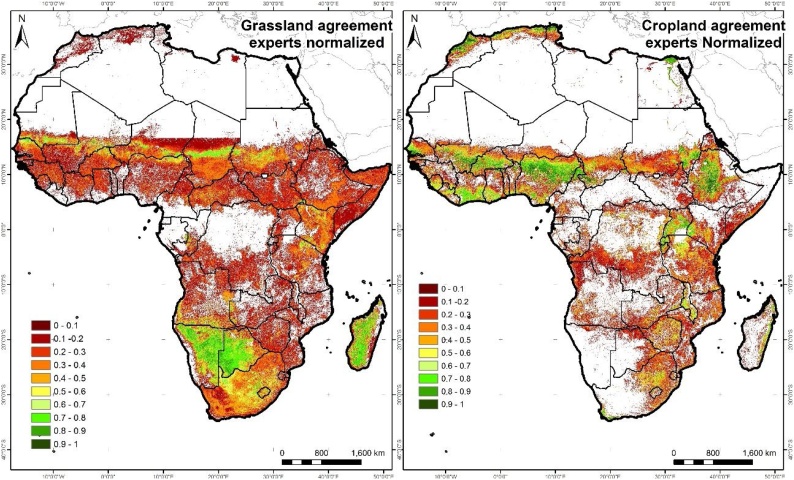
a) Normalised agreement grassland map and b) Normalised agreement cropland map.

The aim of this criterion is to quantitatively capture the expert and regional knowledge of seven research staff members at the JRC Food Security Unit, who have a solid background in agriculture and remote sensing, and in-depth knowledge of the African landscape. To achieve this, an accuracy assessment is carried out using an independent dataset developed for this specific scope, and two overall accuracy measures (OA cropland, OA grassland) are used as input to the criterion. The rationale behind this is to favour the sampling density in areas that are more problematic or difficult to classify. To do this, two probability maps are derived (cropland, grassland), based on the disagreement maps from a Boolean or crisp comparison (Herold et al., 2008), which are normalised to avoid differences due to the different number of datasets per country (Fig. 2).

Reference samples are collected using a random probability proportional to size (PPS) method (Rosén, 1997) that favours sampling in areas of high probability of misclassification. Hence, more reference samples are selected in the areas that tend to disagree on the probability of presence of cropland or low cropland (Fig. 2). In this way, they favour error-prone areas, which are re-sampled at a higher rate. To guarantee that all the countries are represented, a minimum of 10 samples per country are selected. The sampling unit is a homogenous cluster of 3 × 3 pixels (i.e. pixel size 250 m) to reduce the impact of geolocation errors (Congalton and Green, 2009. A total of 1,750 samples are distributed over Africa, of which half are selected from the disagreement cropland map and the rest from the grassland map.

Each expert photo-interprets 250 coded samples that can be superimposed on Google Earth through a. kmz file, and supports the decision using Google Earth Engine to visualise the NDVI profiles for each specific sample. The experts are then requested to indicate the percentage of croplands and grasslands according to four categories: 0 % (absence), 1–25 %, 26–50 % and >50 %. Finally, contingency matrices are computed, considering croplands or grasslands where occupancy is greater than 50 % of the sample.

#### 3.3.2 Construction of standardised performance matrix for each country

**Fig. 3:**
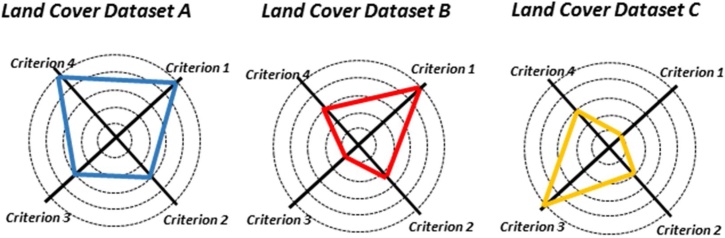
Land cover datasets radar diagrams are an illustrative example for one country. In the radars, it can be observed that the three land cover datasets are very similar with respect to criterion 4. The figure also shows that C scores highest for criterion 1, but lowest for criterion 3. Hence, we can conclude that none of the land cover datasets are better than the others for all criteria.

In order to evaluate each land cover dataset with regard to others, a performance decision matrix is created, based on a set of *n* criteria and *m* alternatives (i.e. land cover). Raw measurements are converted into standardised measures to achieve the standardised performance matrix. This performance matrix makes it possible to compare, on the same scale, how all the land covers behave under each of the criteria. The visual representation of this matrix is shown in Fig. 3.

#### 3.3.3 Sensitivity analysis and ranking

The next step in the MCA consists of creating a weighted regression matrix, using a score that encompasses all the criteria. To achieve this, an additive weighted score (WS) is proposed as follows:

(4) $$WS=\sum_{j=1}^{n}w_{j}\times {criterion}_{ij}$$

in which the final value of a land cover (WS) is computed by the sum of all criteria, with *j* varying between 1 and *n* (number of criteria), multiplied by the weight of each criterion *w,* where 0 ≤ *w* ≤ 1 and ∑ *w*_j_  = 1 (the criterion weights range from 0 to 1 and the sum of all the criterion weights must be 1).

**Fig. 4:**
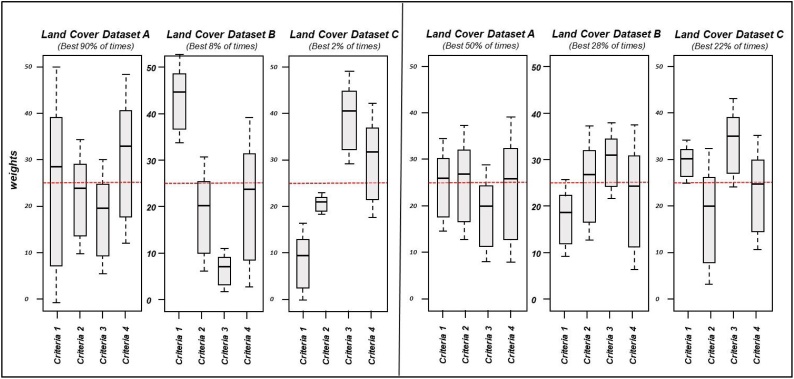
Abridged example of the multiple boxplot distribution of weights from the sensitivity analysis process for two hypothetical situations (countries). In the situation shown on the left, for 90 % of the random weights simulations, the best alternative is Land Cover Dataset A, and only for 2 %, the best land cover dataset is C (and this only occurs with fairly extreme weight values). In the situation shown on the right, the best alternative is Dataset A in 50 % of the simulations, and the other 50 % of probability of being the best alternative are distributed between Dataset B and Dataset C. If the boxes are wide, this means that the land cover dataset is best for a wide range of weights for that criterion. The dashed horizontal red line shows the reference value for the weights, if all the criteria are considered equally important (in the example shown, as four criteria are considered, each one would have the value 100/4 = 25 %). A boxplot above the red horizontal line indicates that the corresponding criterion needs to be weighted above equity to make that land cover the best alternative. Therefore, the extreme position of the boxplot in relation with the red horizontal line is an indication of how good or bad a land cover is in relation to each of the criteria. Specifically, if for one specific land cover, one boxplot takes values significantly higher than the red horizontal line, this is indicative that the criteria corresponding with that boxplot scores higher than other criteria with boxplot under the red line.

Weighting is not easy because there is no objective evidence that one criterion is more important than another. Weights are mostly determined through methods that entail a certain degree of subjectivity. In this research, we address this possible shortcoming by designing a new approach for criterion weighting based on simulating different weights and investigating their relative impact on the resulting outcome or sensitivity analysis. To implement this, thousand iterations of the MCA are run, with weights for each criterion randomly generated from a uniform distribution over the interval [0, 1]. The outcome is the probability for each land cover dataset of being the winner, as well as the distribution of the weights that make a land cover the best alternative (Fig. 4).

Sensitivity analysis illustrates that human intervention is greatly reduced, as in many cases there is one land cover that clearly outscores the rest. Hence, for another land cover to become the best alternative, unbalanced weights should be assigned to most of the criteria considered. In other cases, when more than one land cover dataset seems to be a possible alternative, the human expertise, supported by partial results from the MCA, helps to select the best option to integrate the hybrid map among only the best-rated options (Appendix A includes a real case example for more details).

### 3.4 Accuracy assessment of the resulting hybrid masks

The uncertainty of the resulting hybrid maps, I-Cropland and I-Grassland, is evaluated using a Boolean or crisp comparison with other publicly available datasets, to determine how reasonable our mask results are compared to other maps. I-Cropland is assessed against the latest hybrid initiative in Africa, he Unified Cropland Layer 2014 (UCL 2014), developed under the Stimulating Innovation for Global Monitoring of Agriculture (SIGMA) project at 250 m (Waldner et al., 2015). The data are also evaluated against the previous hybrid land cover mask produced by the JRC at 250 m (MARS-JRC; Vancutsem et al., 2013). I-Grassland comparison is simplest, due to the lower volume of comparable hybrid grassland data available. The I-Grassland mask is compared with the FAO GLC-SHARE grassland layer at 1 km (Latham et al., 2014). Furthermore, the consistency of the resulting mask is evaluated against FAO statistics.

Finally, an accuracy assessment of the I-Cropland mask, UCL 2014 and MARS-JRC, is performed based on metrics derived from the contingency matrix i.e. F-score and overall accuracy, using 6,925 samples from the SIGMA dataset as reference data. The SIGMA data are specifically designed for cropland map validation, and consist of 300 × 300 m localisation. Each sampling unit shows the percentage of cropland, ranging from 0 absence of cropland to 100 % (Laso Bayas et al., 2017). In order to compare the three datasets against the reference samples, SIGMA is converted to a binary legend composed of two categorical classes, cropland (>50 %) and non-cropland (<50 %).

## 4 Results

### 4.1 I-Cropland mask

**Fig. 5:**
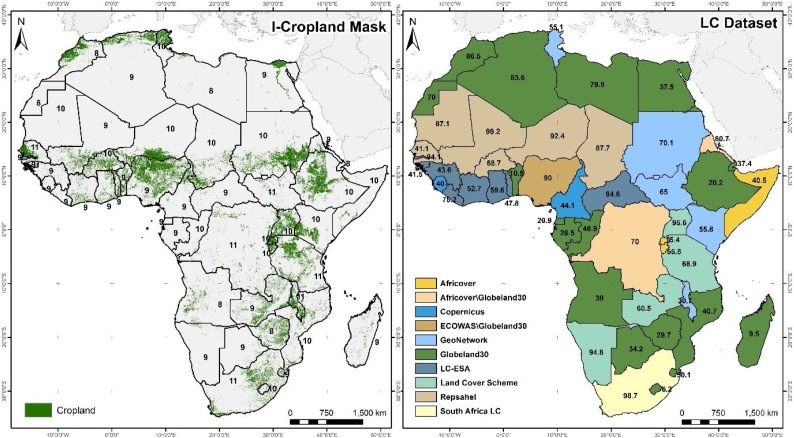
a) I-Cropland mask resulting from application of the MCA; the number of datasets available and that have been analysed for each country are displayed on the map. b) Winner dataset and the probability of winning.

Fig. 5a illustrates the I-Cropland mask created by hybridisation, whereas 5b shows the land cover datasets chosen and the probability of winning with the MCA. It is essential to underline that after running the MCA, the representativeness of the best-rated land cover datasets for each country is assessed based on a visual inspection of high spatial resolution imagery from Google Earth, in combination with the partial results derived from the MCA (i.e. spiders, box, ranked), and modified when required. Thus, the solution ranked first is not automatically selected as the best land cover dataset. For instance, in 10 % of the countries, other datasets with lower rankings (but still among the highest scoring alternatives) are integrated into the final I-Cropland mask. For example, in Togo, GlobeLand30 (with 10.9 %) is preferred to ESA S2 (with 32.5 %) because the latter represents cropland inappropriately based on visual inspection. The resulting I-Cropland illustrates well-known patterns of cropland in Africa, including the Sahelian band, the surroundings of the Great Lakes, the northern coast, along the Nile Basin and in southern Africa. Regionally produced land cover datasets generally capture cropland distribution well, as this approach is adapted to the specificities of each region and due to regional expertise involved in the production process (Song et al., 2014a,b). However, the differences between regionally produced land cover datasets and global products tend to decrease with an increase in spatial resolution of the input satellite data, such as CGLS-LC100 (100 m). Our findings show that global datasets can overcome regional alternatives, as in Ethiopia, where they perform (20 %) better using the MCA compared to regional (and older) products such as the World Bank or Land Cover Scheme. These new datasets prove very useful in countries where there is a widely recognised lack of regionally produced land cover information, as in Namibia or Angola (See et al., 2015b).

Similar to previous findings (Pérez-Hoyos et al., 2017), GlobeLand30 appears to be suitable for monitoring croplands, especially in arid zones. In fact, according to the sensitivity analysis, GlobeLand30 is among the best-rated alternatives for almost all the countries. Our findings confirm the expectation that high spatial resolution is one of the key attributes to reliably capture the earth’s surface, and consequently to accurately classify croplands. On the contrary, low spatial resolution data, such as GLC2000 and GLCNMO2008, barely resulted in a viable option, because cropland detection at such resolution is penalised due to misclassification of cropland with similar spectral classes such as grassland.

Timeliness is not always considered one of the crucial determinants, and in some countries GlobeLand30 (2010) stands out compared to the ESA S2 prototype (2016) or GCLS-LC100 (2015). Indeed, the ESA S2 prototype derived from Sentinel 2 was quite unsuitable for the areas in the sub-Sahelian zone. This is in line with previous findings that ESA overestimated cropland in Sudan and underestimated it in Morocco and Algeria (Lesiv et al., 2017). Moreover, in some cases older data such as Africover remain adequate to represent croplands, as in Somalia (40 %).

There are zones with low probability scores, i.e. around 50 %, mainly in Western Africa (e.g. Ghana, Sierra Leone and Liberia). These areas are challenging to map by remote sensing because of the low agricultural intensification, high variability of agricultural systems with more than one season, and presence of clouds that hampers classifications (Vitrou et al., 2012). Moreover, in Western Africa there is frequent presence of oil palm and forest canopy in agricultural parcels, which is not relevant for food security early warning. Due to the presence of trees and differences in legend definitions, cropland overestimation occurs in some cases when compared with the statistics. In addition, these errors are translated from one dataset to another, as occurs between GlobCover and LC-CCI 2015.

Finally, there are particular instances in which two datasets appear to be spatially complementary, as occurs in the Republic of Congo where Africover has the highest probability of winning (54.9 %), while GlobeLand30 is the second option (15.4 %). After a meticulous analysis, we realised that the timeliness was responsible for the expansion of croplands. A similar situation occurs in Nigeria, where GlobeLand30 scored high (80 %) for almost all the criteria but important cropland areas were missing, mainly in the western part of the country. In this case, it was complemented by ECOWAS (12 %); however, the main constraint was under the low spatial resolution criterion. In this particular case (i.e. Eritrea, Nigeria, DRC), the first two rated land cover data are merged.

### 4.2 I-Grassland mask

**Fig. 6:**
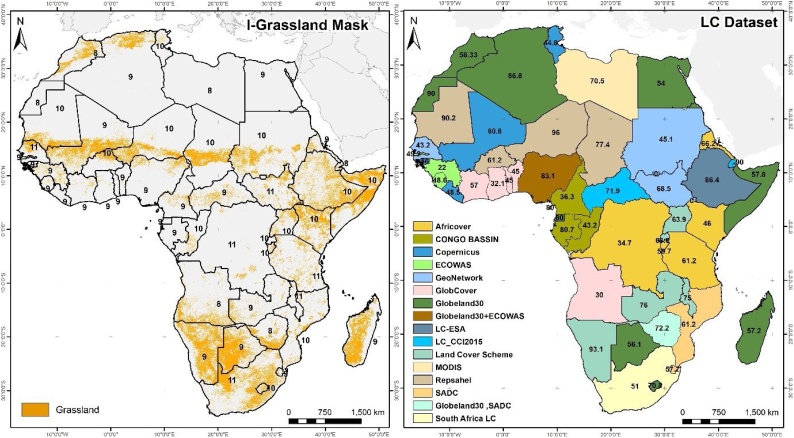
a) I-Grassland mask resulting from application of the MCA; the number of datasets analysed are displayed for each country. b) Winner dataset and the probability of winning.

Fig. 6a gathers the best performing products based on the MCA with a thorough revision of results, while 6b shows the source used for each country. Development of the I-Grassland mask was more difficult, as it is recognised that classifying grassland is a challenge due to its high level of confusion with other classes, such as forest or shrubland, especially in savannah ecosystems (Tsendbazar et al., 2018). The probability values are generally lower than in the ICropland mask, and based on results, more than one dataset is closer to the ideal solution for some countries. The highest probability values are reached in countries such as Mauritania (90.2 %) and Niger (96 %), where regional initiatives such as Repsahel clearly outperform other products, while the lowest values are reached in Angola (30 %), Ghana (32.1 %) and Guinea (22 %).

Most of the surface of the grassland mask is situated in southern Africa, followed by the Greater Horn of Africa and the Sahelian band. As expected, products derived from higher resolution sensors (e.g. GeoNetwork, Land Cover Scheme) contribute substantially more than other products to the I-Grassland mask. However, low spatial resolution products such as GlobCover and LC-CCI 2015 appear as possible solutions to representing grassland in the Central African Republic or in Togo. In this case, the timeliness criterion plays an important role that disqualified several datasets for selection regardless of its goodness. For example, in Zimbabwe the SADC is spatially combined with GlobeLand30, as the latter complements the product in areas where an expansion of grassland has occurred in the last ten years.

The mask displays some spatial inconsistencies arising from the border effect, as occurs in north-western Nigeria. The mask has been generated on a per-country framework that is appropriate in national-level early warning systems such as ASAP (Rembold et al., 2019), so the border effect cannot be completely avoided. To overcome this issue, some modifications to the proposed method could be considered, such as applying MCA on a per pixel basis (Pérez-Hoyos et al., 2012a,b).

### 4.3 Accuracy assessment of the resulting hybrid masks

Based on the 6,925 sample pixels from SIGMA, the overall accuracy OA and the F-score for I-Cropland, MARS-JRC and UCL for Africa are assessed. Although the results should be interpreted carefully, due to the low number of samples for some countries (e.g. Burundi, Rwanda), this remains an effective manner to compare each dataset with another. Generally, results reveal that I-Cropland is mapped with higher accuracy in comparison to other datasets (see values in Appendix B). UCL presents closer accuracies to our hybrid map than that displayed by MARS-JRC, with better accuracy for some countries such as Tunisia, where OA for UCL (0.83) is higher than for I-Cropland (0.71). However, in this specific case, the assessment is biased due to semantic discrepancies. This is because the SIGMA definition of cropland, also used in UCL, excludes permanent cropland (Laso Bayas et al., 2017), whereas we include it.

**Fig. 7:**
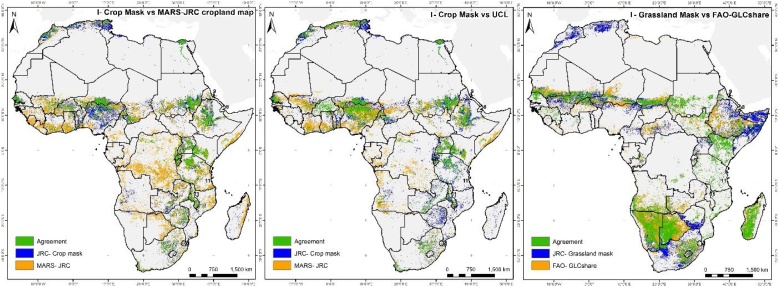
Spatial agreement between I-Cropland mask and MARS-JRC (Vancutsem et al., 2013); I-Cropland and UCL (Walder et al., 2015); and I-Grassland and FAO GLC-SHARE (Latham et al., 2014). Agreement means that our mask and the compared dataset agree (green); blue corresponds to our mask and orange to the compared dataset.

Fig. 7 illustrates the spatial agreement in a pairwise comparison. There are large differences between I-Cropland and UCL, and between I-Cropland and MARS-JRC, for the spatial distribution of croplands, with only 38.1 % and 32.1 % of agreement, respectively. One of the largest areas of disagreement for croplands is mainly located in western Africa, particularly in Nigeria, Benin, Togo and Ghana, where the total amount of croplands is overestimated in the compared datasets. In these countries, our method benefits from the better spatial resolution of newly released products, which allow a more accurate delineation of croplands and probably explain these differences.

One of the main discrepancies between I-Cropland and MARS-JRC is in the Democratic Republic of Congo, where cropland is largely overestimated by the latter when compared with FAO statistics. Our hybrid map is better for Madagascar, where datasets developed before 2010 tend to repeat the same errors, resulting in a misclassification of cropland in southern zones and in an overestimation in eastern zones. There is less spatial inconsistency compared with UCL; I-Cropland appears to better identify crops in Angola, CAR, Mauritania and Chad, where UCL overestimates. These results are also supported by a comparison with the statistics and the accuracy assessment (Appendix B). Discrepancies in Zimbabwe correspond to an overestimation in GlobeLand30; the selected data that feeds into our hybrid map (see Fig. 7). This is one of the few countries where the F-score for I-Cropland (0.885) is lower than the UCL F-score (0.951).

The I-Grassland mask shows higher agreement with FAO GLC-SHARE (43.31 %) than the crop mask. In general, the overall pattern of I-Grassland is quite similar to the comparison layer because in some countries (e.g. Sudan and South Sudan), GLC-SHARE uses the same input datasets, inevitably leading to innate agreement. The main discrepancies are mostly in North African countries (e.g. Morocco), where the newest datasets such as GlobeLand30 turn out to be the favourite solution for our mask.

## 5 Discussion

Since the first JRC crop mask for Africa was published in 2012 (Vancutsem et al., 2012), using a combination of multiple global and regional products, new datasets have emerged that better satisfy the continuous need for high quality agricultural information. Detailed and accurate estimates of cropland and grassland are highly relevant for food security-related issues and for baseline information for early warning systems, such as the recently launched JRC ASAP system (Rembold et al., 2019). However, existing land cover datasets disagree with each other due to differences in classification scheme, spatial resolution, methodology and sensors used, among other factors.

The hybridisation approach, as applied in this research, offers an alternative for developing a better dataset in a quasi-automatic and cost-effective way. Previously fused maps mainly focus on cropland (Fritz et al., 2010) or forest (Schepaschenko et al., 2015), but rarely explore the integration of grassland that is capital in agricultural production for food security. In this paper, we explore the suitability of a semi-automatic method based on an MCA for hybridisation, due to its simplicity and intuitive logic which make it transparent and completely objective. The comprehensive nature of the method provides a ranking that can easily be traced back, as well as the known cause as to why a dataset is more or less appropriate for a given purpose. For example, in Eritrea, the use of Africover is restricted under the timeliness criterion, because from the year of production (1995) to the nominal year of our masks (2016), cropland surface has increased in the country. Undoubtedly, this is not the first attempt to hybridise data using MCA (Waldner et al., 2016), but the work benefits from the inclusion of new datasets such as LC-CCI 2015, ESA S2 prototype and CGLS-LC100. Results take into account that land cover datasets derived from high resolution satellite data are useful for better characterisation of highly fragmented landscapes such as those found in Africa, and in a considerable number of countries. GlobeLand30 and S2 prototype resulted as the ideal solution. Moreover, these latest datasets considerably improve characterisation of countries such as Namibia or Chad, where information is scarce (See et al., 2015b). In fact, compared with other approaches where the use of regional datasets were prioritised (Walder et al., 2015; Vancutsem et al., 2013; Latham et al., 2014), our results suggest that the selection of high resolution global datasets is a good alternative and sometimes even better than regional initiatives (e.g. Zimbabwe).

The MCA approach presented in this study guarantees flexibility and can be easily modified to accommodate new datasets or criteria in case the purpose of the mask changes. For example, it could include a criterion related to water supply (irrigated, rainfed) in the event that interest focuses more on the contribution of irrigated crops to national production. The method allows simultaneous evaluation of a huge number of datasets. One of the biggest benefits of this research is in how it deals with weights in the multi-criteria analysis. Usually, weights are uniform for all the criteria (Waldner et al., 2016), or the weighting process is based on expert knowledge that involves human intervention and then some subjectivity (Munda et al., 1994). This research tackles this issue by means of a fully objective weight method, based on random weights, followed by a sensitivity analysis to investigate the response of the different land cover datasets once the relative importance rating is modified. Nonetheless, there are still several issues to be addressed and taken into account in the method developed here. One of the overriding concerns lies in legend harmonisation, particularly the definition of grassland. Here, we focus only on herbaceous cover, because in the automatic detection of hotspots carried out in ASAP (see more details in Rembold et al., 2019), the interest is in having a clear spectral signal. However, it is well known that livestock also browses woody savannahs, this area being strategic for guaranteeing food supply. Another consideration concerns the process of hybridisation itself, which assumes that at least one against another. However, there are zones that are prone to error, such as western Africa, in which greater effort is still needed to accurately classify agriculture (See et al., 2015b).

Even if there is still room for improvement, the methodology proposed here offers a good strategy to combine different land cover products, resulting in better cropland and grassland masks when compared with previous attempts, statistics or based on an independent accuracy assessment. We consider that the resulting mask is the best possible in view of current sources available, and for our specific purpose. The rising number of initiatives dedicated to enlarging the volume of reference data belonging to a particular class such as cropland or grassland (e.g. SIGMA), coupled with the new release of quality statistical data (FAO, 2017) are expected to overcome some of the issues with our method.

The ensemble approach proves suitable for addressing user-specific requirements by adapting existing thematic land cover information and creating a user-tuned map. For instance, cropland classes are not relevant for users who are only interested in forest applications. In this context, hybridisation proves key to exploiting the strengths of individual land cover datasets, especially now when continuing progress in remote sensing technology is underpinning the production of quality land cover maps at higher spatial and temporal resolution.

## 6 Conclusion

The JRC early warning decision support system ASAP (Anomaly hot Spots of Agricultural Production) aims to trigger warnings about anomalies in agricultural production (i.e. cropland and grassland) that require essential input information on agricultural land use. Nonetheless, in a preliminary investigation (Pérez-Hoyos et al., 2017), considerable discrepancies were found among data, and a clear preference of one land cover product over another is still questionable. To overcome this issue, our research proposes a methodological semi-automatic framework relying on multi-criteria analysis to integrate different land cover datasets, to derive two hybrid static masks (cropland and grassland) for Africa, at 250 m spatial resolution and for the nominal year 2016.

Results demonstrate the capability of MCA to capture agricultural information over large areas, when the two outcomes are compared to other existing individual maps used as benchmarks (e.g. FAO GLC-SHARE). Our mask improved results for several error-prone countries, such as Madagascar, and was able to highlight problematic areas where further efforts are needed in the future due to lack of accuracy of the datasets analysed. The MCA was easily reproducible and integrated quantitative criteria to select data in a transparent and flexible way. Moreover, the method was designed to handle large datasets and was able to provide traceability as to which one is better than the other and why. The development of fusion approaches, such as the one conducted here, is highly relevant due to the steady incorporation of emerging land cover datasets. These methods are essential given the forthcoming increase in sources due to the open availability of high resolution imagery (Landsat 8; Sentinel 1 and 2), coupled with the development of applications such as Google Earth Engine.

The integrated agricultural masks are freely accessible at https://mars.jrc.ec.europa.eu/asap/download.php. The dataset on the webpage is a degradation to 1 km spatial resolution from the mask obtained in this research. Data values correspond to an area fraction that represents the percentage of the pixel occupied by cropland and grassland (scaled from 0 to 200 %). The 250 m dataset are available upon request at jrc-asap@ec.europa.eu.

## Figures and Tables

**Table:** 

**Table:** 

## Appendix A Real example of the sensitivity analysis process

Example of the multiple boxplot distribution of weights derived from the sensitivity analysis process for a) Chad and b) Angola for cropland. In Chad, for 87.7 % of the random weights simulations, the best alternative is Repsahel and only for 2.5 %, the best land cover dataset is Copernicus (this only occurs with extreme weight values). In Angola, the best alternative is Copernicus in 41 % of the simulations and GlobeLand30 in 39 %.

The red dashed horizontal red line shows the reference value for the weights, if all the land covers are considered equally important (in the example, each criterion would have 100/6 = 16.6 %). A boxplot above the red horizontal line indicates that the corresponding criterion needs to be weighted above equity, for the land cover to be the best. Therefore, the position of the boxplot compared with the red horizontal line is an indication of how good or bad a land cover is in relation to each of the criteria. Where the boxplot has values higher than the red horizontal line, it means that the land cover scores higher for that criterion.

The results of the sensitivity analysis greatly reduce human intervention. As occurs in Chad, one land cover is clearly better than the rest, since for another land cover to become the best alternative, unbalanced weights should be assigned to most of the criteria considered. Therefore, intervention by experts is unnecessary. On the other hand, the sensitivity analysis shows that more than one land cover dataset could be considered the best alternative to be integrated into the mask. Consequently, the expert can choose among the different alternatives based on the result of the simulation. In this context, it is not necessary to propose weights for each criterion, as the analysis is mainly based on the boxplot.
